# The Relative Nature of Perception: A Response to Cañal-Bruland and van der Kamp (2015)

**DOI:** 10.1177/2041669515599898

**Published:** 2015-09-02

**Authors:** Sally A. Linkenauger

**Affiliations:** Psychology, Lancaster University, Bailrigg, Lancaster, UK

**Keywords:** visual perception, affordance, ecological, distance perception, size perception

## Abstract

Cañal-Bruland and van der Kamp present an argument about the incommensurate relationship between affordance perception and spatial perception in a criticism of Proffitt and Linkenauger’s phenotypic approach to perception. Many of their criticisms are based on a difference in the interpretation of the core ideas underlying the phenotypic approach. The most important of these differences in interpretations concern fundamental assumptions about the nature of the perceptions of size and distance themselves. Extent perception must be relative to the organism; therefore, there can be no veridical perception of space. Also, we argue in the phenotypic approach that space perception is an emergent property of affordance perception; they are not different types of perceptions as Cañal-Bruland and van der Kamp presume. Third, affordance perception need not be perfectly accurate, just good enough. Additionally, affordance perception need not be dichotomous; this presumption likely originates in the methodology typically employed to study affordance perception. Finally, I agree with Cañal-Bruland and van der Kamp that joint research efforts will clarify and improve our understanding of these issues.

I applaud Cañal-Bruland and van der Kamp (2015) for their attempts to reconcile different approaches and for suggesting to combine our efforts to move forward as a science. However, Cañal-Bruland and van der Kamp (2015) and I differ in our interpretation of several critical points in Proffitt and Linkenauger (2013), which are crucial to the phenotypic approach to perception. Here, I go through the five steps introduced in Cañal-Bruland and van der Camp (2015) to address each one according to my interpretation of their concerns through the lens of how I have conceptualizing both perception and perceptual metrics to date.

## Step 1: A Salute to Gibson!

Gibson (1986/1979) provided a wonderful blueprint about how we should think about perception in terms of an ecologically adaptive process that occurs over time. Hence, the purpose of perception from an ecological standpoint is to discover the opportunities for action (affordances). However, the authors draw a distinction between affordance perception and the perception of spatial layout. They seem to support the view that affordance perception can be separated from the

perception of the spatial world such as described by physics in terms of metric units of space and time, and which conceives the world as is independent of the observer. Of course, we can perceive spatial properties … this is a different type of perception. (p. 64)

I disagree. Gibson also doubted the veracity of this assumption.

Children learn to see sizes in terms of prehension: they see the span of their grasp and the diameter of a ball at the same time. Long before the child can discriminate one inch, or two, or three, he can see the fit of the object to the pincer-like action of the opposable thumb. The child learns the scale of sizes as commensurate with his body, not with a measuring stick. (Gibson, 1986/1979, pp. 234–235)

To me, the conception that the spatial layout is or can be perceived independently of the observer has to be incorrect. This is one of the main ideas communicated through Gibson’s work. The only thing out there in the physical world is information that the organism learns is meaningful relative to its own abilities. Hence, everything is relative to the organism. A table is only solid because it is denser than one’s hand. Up is only up because gravity is strong enough to anchor us to the ground. Consequently, I think there are a few terms that need to be stricken from our vocabulary when discussing distance and size perception, including but not limited to, veridical, real, accurate, true, and absolute. In terms of space perception, these adjectives do not exist.

To illustrate, does Gulliver perceive a boulder to be the same size as a Lilliputian? The obvious answer is no; the Lilliputians perceive the boulder as much larger than Gulliver. However, if so, then who perceives the veridical size? Gulliver or the Lilliputians? I am of the opinion that no one sees the veridical extent because veridical does not exist. Extents are always perceived as relative to the organism.

Every extent derives its meaning from the metric to which it is scaled. I argue that it is not veridical or accurate or absolute that matters in terms of metrics. What matters is that the metric must be meaningful for it to be useful. The only thing meaningful about a size or distance is that it limits the number of actions that can be performed on or over it. Thus, logic demands that this metric is derived from the body. Telling someone that an object is two widgets wide provides absolutely no useful information. However, if I tell the same person that a widget is their foot length, I will have provided useful information about that object’s size by anchoring it to the body.

## Step 2: Affordance Perception Is Usually Reflected in Measures of Spatial Perception!

The authors mistakenly assert that affordances are rarely measured when assessing their effects on spatial perceptions. In the majority of my research, I use affordance measurements in addition to spatial measurements (Lessard, Linkenauger, & Proffitt, 2009; Linkenauger et al., 2014; Linkenauger, Leyrer, Bülthoff, & Mohler, 2013; Linkenauger, Witt, Stefanucci, Bakdash, & Proffitt, 2009; Linkenauger, Witt, & Proffitt, 2011). Others do as well (Eves, 2014; Eves, Thorpe, Lewis, & Taylor-Covill, 2014; Taylor, Witt, & Sugovic, 2011; White, Shockley, & Riley, 2013). Most of these studies show the hypothesized relationship between changes in affordance and size or distance perception.

## Step 3: Affordances Need not be Veridical or Dichotomous! A Salute to Darwin!

We proposed in Proffitt and Linkenauger (2013) that the extent over which an action can be performed is used as a perceptual metric. Consequently, it is unclear how dichotomous affordance judgment and continuous spatial judgments presents a problem. The distance over which an action can be performed is continuous; this is the scale. The metric is an emergent property of the affordance.

The authors also argue that “affordance perception must be veridical; otherwise, perception would not be adaptive and animals could not survive (p. 64).” They use this assumption to contrast with spatial perceptions that are typically characterized by systematic distortions. First, affordance perception does not need to be anywhere near veridical; it only needs to be good enough. Evolution is a satisficing system, not an optimizing system. We are only as good as we need to be; not the best that can be. Much research has shown that people are not completely accurate in assessing their action capabilities, even on tasks on which they are experts (Weast, Shockley, & Riley, 2009). People also misperceive their affordances on a daily basis. Countless times I have attempted to reach a glass that was beyond my reach or tried to grasp an object that was too big. I am not perfect, but I am good enough to amble about in my environment.

Consequently, affordance perception does not need to be (nor is it) dichotomous; this dichotomy is a by-product of the way we design studies to measure it. Whether individuals attempt to perform the action is merely a way to indirectly measure affordances, but it is unlikely to be a direct measure. The closer individuals get to their action boundaries, the more variability in these dichotomous measures emerge, which is likely indicative of the probabilistic nature of perceptions of affordances (this idea is introduced and supported in Franchak & Adolph, 2014). I doubt there is a distinct threshold that people experience at the boundary of their capabilities. One interesting area for future research is how we determine the location of the perceived action boundary if it is a probability distribution.

## Step 4: Which Direction Next?

Admittedly, I do not know why changes in spatial perception and affordance perception are not of the same magnitude despite them always changing in the same direction. Firestone (2013) makes a good argument here. However, this argument does nothing to disprove the phenotypic perspective, but rather uncovers directions for future research. These differences in effect size could be a by-product of the different measures used to assess affordance and distance perceptions. Alternatively, affordances could be nonlinearly mapped onto spatial percepts. For instance, the probabilistic function associated with affordance perception introduced by Franchak and Adolph (2014) could be used as a possible transform of the perceptual metric.

Cañal-Bruland and van der Kamp (2015) suggest that perhaps spatial percepts only slightly above and below the action boundary are influenced by changes in action capabilities; otherwise, they are perceived veridically. However, I have a hard time conceptualizing the proposed explanation because I do not understand what veridical is. Additionally, previous research has shown that extents outside of the action boundary are not influenced by manipulations of action capabilities; whereas, extents within the action boundary are influenced by these manipulations (Lessard et al., 2009; Linkenauger, Witt, & Proffitt, 2011).

## Step 5: Collective Efforts! Count Me In!

Sure, it is indeed possible (even likely!) that some of these effects are spurious. Over the years, my colleagues and I have run hundreds of studies. Just by chance alone, some of these findings will not be replicable. I would be happy to be included in a replication attempt to parse apart the effects that persist and those that do not. Overall, though, many will not accept this approach regardless of what we find, and however, many experiments support our hypotheses. Hence, I would rather move forward and find out more about how we experience the world around us.

As we explicitly stated in Proffitt and Linkenauger (2013), we are pretty sure that we are wrong about many things. We are completely fine with that. Finding out that some ideas are wrong is one of the only ways to learn something new and move forward. Some of the work done by Bingham, Pan, and Mon-Williams (2014) has shown support for calibration acting as a perceptual metric, which may also redefine the ways we think about perceptual scaling (also see Pan, Coats & Bingham, 2014). I am looking forward to what future experimentation may uncover.
